# Subretinal peripapillary biopsy-proven sarcoidosis: a case report

**DOI:** 10.1186/s40942-022-00412-1

**Published:** 2022-09-03

**Authors:** Jason R. Daley, Svetlana Cherepanoff, Peter G. Heydon, Adrian T. Fung

**Affiliations:** 1 Liverpool Hospital, Sydney, NSW Australia; 2 The University of Sydney, Sydney, NSW Australia; 3 Sydpath, St Vincent’s Hospital, Sydney, NSW Australia; 4 St Vincent’s Clinical School, University of New South Wales, Sydney, Australia; 5 Westmead and Central Clinical Schools, Specialty of Ophthalmology and Eye Health, The University of Sydney, Sydney, NSW Australia; 6 Department of Ophthalmology, Westmead Hospital, Corner of Hawkesbury and Darcy Roads, Westmead, NSW 2145 Australia; 7 Department of Ophthalmology, Faculty of Medicine and Health Sciences, Macquarie University Hospital, Sydney, NSW Australia

**Keywords:** Sarcoidosis, Subretinal, Optic nerve granuloma, Vitrectomy

## Abstract

**Background:**

To report a case of a subretinal, unilateral, peripapillary granuloma that was diagnosed as sarcoidosis by a 27-gauge pars plana vitrectomy subretinal biopsy. Sarcoidosis is a chronic idiopathic granulomatous inflammatory disease, that has ocular involvement in 10–80% of patients. It is often mistaken for many other primary ocular diseases because the condition can involve any structure in or around the eye. Previous case reports of peripapillary sarcoidosis have either been limited to the choroid or presented with additional ocular and systemic signs, hence have not required an intraocular biopsy.

**Case presentation:**

A 54-year-old Filipino male presented with a 6-month history of painless blurred vision in his right eye. Fundus examination revealed a large white peripapillary lesion. Enhanced-depth imaging optical coherence tomography confirmed the subretinal location of the mass. Indocyanine green angiography demonstrated absolute hypofluorescent blockage with satellite lesions. A whole-body positron emission tomography scan demonstrated widespread lymphadenopathy, but investigations including an inguinal lymph node biopsy were inconclusive. Following growth of the peripapillary lesion and worsening vision, a 27-gauge pars plana vitrectomy subretinal biopsy was performed which confirmed sarcoidosis. He was treated with oral corticosteroids and transitioned to long term immunotherapy with methotrexate.

**Conclusions:**

Sarcoidosis can present in the subretinal space, around the optic nerve without other ocular findings.

## Background

Sarcoidosis is an idiopathic, systemic, and granulomatous inflammatory disease. Ocular involvement is relatively common, and is the first clinical manifestation of the disease in 21% of cases [1].

Ocular sarcoidosis can affect any part of the eye, manifesting as a uveitis, scleritis, dry eye, optic neuritis, choroidal granuloma and exophthalmos [2]. Uveal tract involvement is the most common ocular finding and the majority of sarcoid uveitis cases are chronic and bilateral. Herein we describe a rare case of a subretinal, peripapillary granuloma, presenting as the first manifestation of systemic sarcoidosis as diagnosed by 27-gauge vitrectomy subretinal biopsy.

## Case presentation

A 54-year-old Filipino male was referred with a 6-month history of painless blurred vision and a subretinal peripapillary lesion in his right eye. Treatment had been administered elsewhere for a possible inflammatory choroidal neovascular membrane (CNV) with oral prednisone and five injections of intravitreal bevacizumab. This initially led to resolution of the peripapillary subretinal fluid but not the subretinal mass. The subretinal fluid recurred as the prednisone was tapered. He had no significant ophthalmic history and fundus photographs taken by his optometrist, two years prior were normal. His past medical history included type 2 diabetes mellitus, hypertension, hyperlipidaemia, left herpes zoster ophthalmicus (without ocular involvement), steatohepatitis and obesity with previous gastric sleeve surgery.

**Fig. 1:**
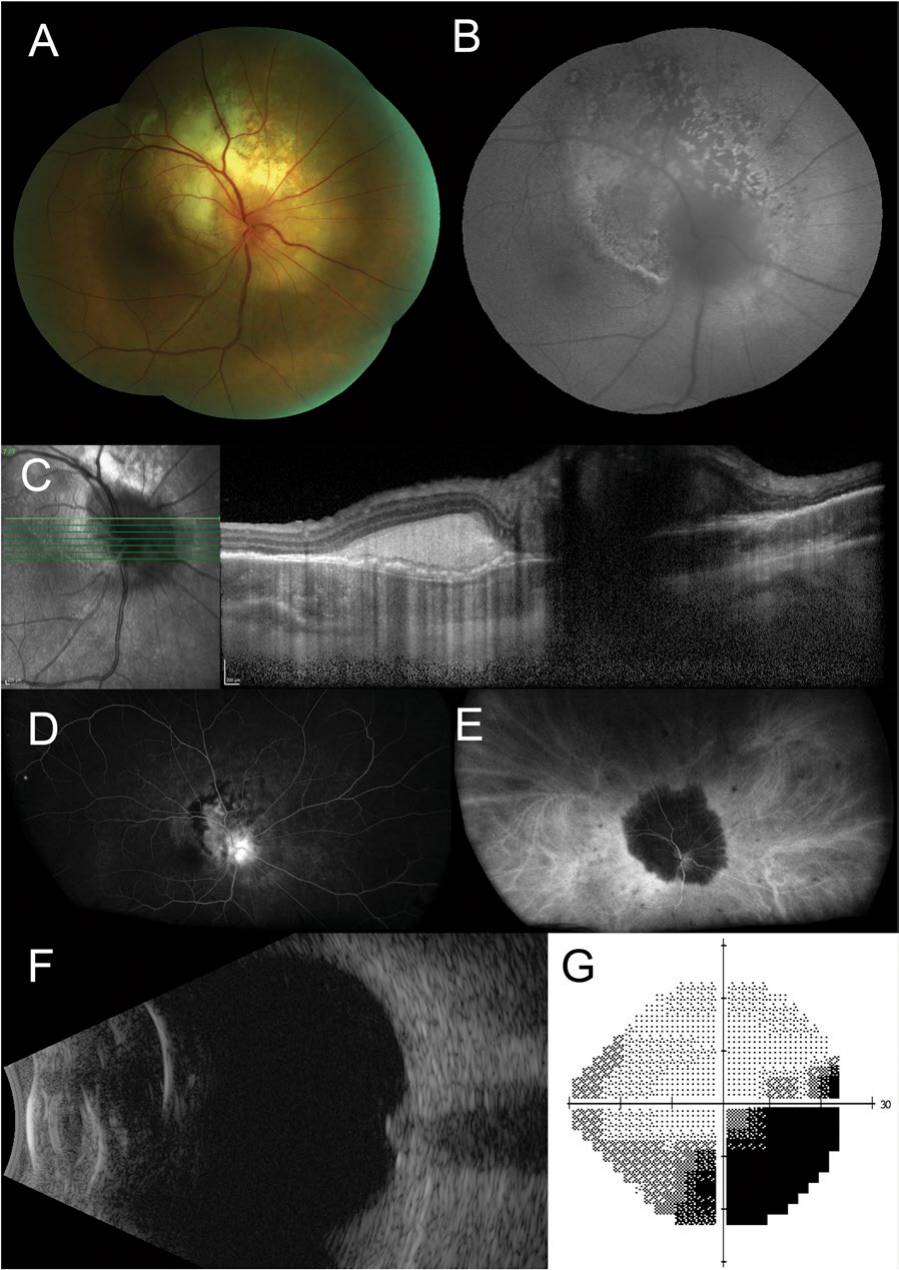
**A** Color fundus photograph of the right eye reveals a large, white, subretinal lesion centred on the optic nerve head. **B** Fundus autofluorescence shows a speckled picture. **C** Enhanced depth imaging optical coherence tomography scan of the right macula shows a hyperreflective subretinal mass. **D** Fluorescein angiography demonstrates diffuse hyperfluorescence, focused over the peripapillary, subretinal lesion and blocked fluorescence at its margin with no significant leakage. **E** Indocyanine green angiography shows absolute blockage of fluorescence by the lesion with multiple smaller hypofluorescent spots in the peripheral retina. **F** An axial 12 o’clock meridional B-scan ultrasound demonstrates an enlargement of the optic nerve head with no retrobulbar extension. **G** Grayscale map from the Humphrey visual field test using a 24–2 pattern, shows a right inferotemporal scotoma

On examination, best-corrected visual acuity (BCVA) was 20/25 in the right eye, and 20/20 in the left eye. Intraocular pressures were 9 mmHg right eye, 10 mmHg left eye. There were no signs of anterior uveitis, vitritis or retinal vasculitis in either eye. Fundus examination of the right eye revealed a large, white, subretinal lesion, centred on the optic nerve head (Fig. 1A) that demonstrated speckled autofluorescence (Fig. 1B). Enhanced-depth imaging optical coherence tomography (EDI-OCT) showed a hyper-reflective subretinal mass without thickening of the choroid, subretinal fluid or macular edema (Fig. 1C). On fluorescein angiography there was hyperfluorescent leakage of the optic nerve, staining of the peripapillary subretinal lesion and blocked fluorescence at its margin but no signs of retinal vasculitis (Fig. 1D). Indocyanine green (ICG) angiography demonstrated striking absolute blockage of fluorescence by the lesion with multiple small hypofluorescent satellite spots throughout the fundus (Fig. 1E). B-scan ultrasonography revealed an echodense lesion at the optic nerve head (Fig. 1F). 24–2 Humphrey visual field testing showed a right inferotemporal scotoma (Fig. 1G). The left eye was normal.

**Fig. 2:**
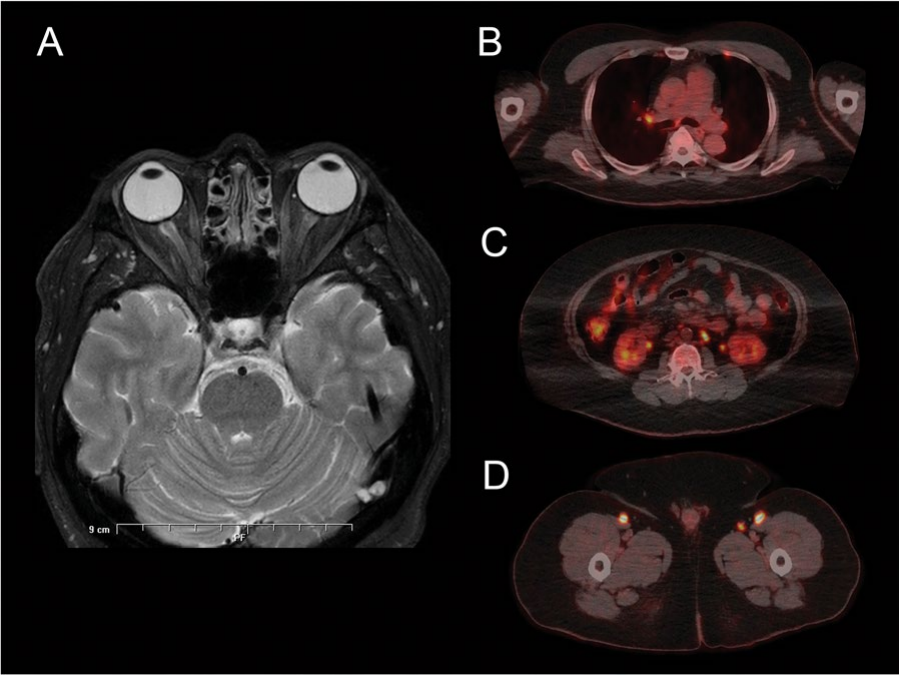
**A** Magnetic resonance imaging of the orbits (axial section, T2-weighted) reveals increased intraneural signal of the right optic nerve. **B** Positron emission tomography (axial section) detects an increased focal 18-Fluodeoxygluocse uptake in the right hilar lymph node. **C** Intense uptake in bilateral para-aortic and left infra-renal lymph nodes. **D** Intense uptake in bilateral inguinal and femoral lymph node regions

Complete blood count, erythrocyte sedimentation rate, QuantiFERON gold testing and an autoimmune screen revealed no abnormalities. Syphilis, Toxoplasma and Bartonella serology were negative. The angiotensin-converting enzyme (ACE) level was taken twice. The first reading was normal at 55 U/L, but a subsequent second test was slightly raised at 82 U/L (reference range: < 81 U/L). The patient was not taking any ACE inhibitors. Magnetic resonance imaging (MRI) of the brain and orbits revealed increased intra-neural signal in the right optic nerve but no optic nerve sheath tumour or glioma (Fig. 2A). Computed tomography (CT) of the chest showed a bulky appearance to the lower oesophagus with an adjacent right retrocrural lymph node noted, thought to be related to previous gastric sleeve surgery. No hilar lymphadenopathy was detected.

The patient was referred to a haematologist due to concern for the possibility of an unusual presentation of an ocular lymphoma or a paraneoplastic process such as paraneoplastic cloudy vitelliform subretinopathy. He underwent a lumbar puncture which showed no malignant cells. A bone marrow biopsy was obtained from his left posterior superior iliac spine, which demonstrated reactive marrow with variable cellularity with mild megakaryocytic hyperplasia and mild dyserythropoiesis. No neoplastic lymphoid population was detected on flow cytometry. Positron emission tomography (PET) showed metabolically active disease in multiple lymph node groups (bilateral pulmonary hila, para-aortic, iliac, and inguinal), multiple bony skeletal sites, the right ventricle and liver (Fig. 2B–D). A core biopsy of the right deep inguinal lymph node showed a non-necrotizing granulomatous lymphadenitis with possible etiology including infection (mycobacteria, fungi, parasites) and inflammation.

Three months later the patient’s vision deteriorated to 20/100 in the right eye and OCT scans showed subfoveal fluid. Due to persisting diagnostic uncertainty, a 27-gauge pars plana vitrectomy and subretinal biopsy was taken from the peripapillary lesion. A posterior vitreous detachment was induced, and the intraocular pressure was temporarily raised to 80 mmHg at the time of biopsy to reduce the risk of haemorrhage. A 25-gauge curved scissor was used to excise the subretinal tissue which was removed through an anterior 18-gauge sclerostomy. A small sectoral retinal pigment epithelium tear developed superotemporal to the optic disc and the biopsy site was surrounded with endolaser retinopexy. Cryotherapy using triple freeze thaw technique was applied to the sclerostomy site, to prevent seeding of potentially malignant cells. Intravitreal silicone oil (1300 centistoke) was used for tamponade.

**Fig. 3:**
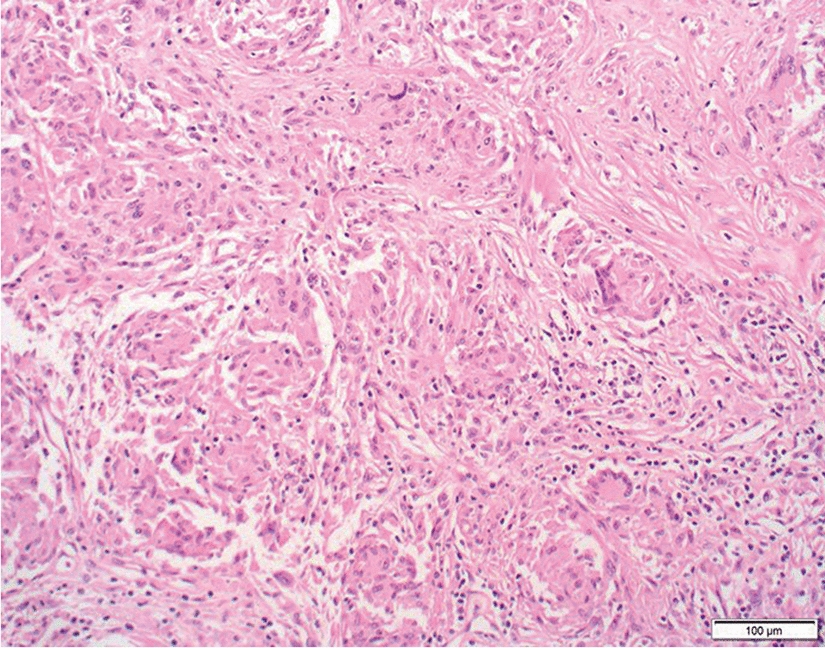
Cell block Hematoxylin and Eosin section of the right subretinal biopsy (× 200 magnification) shows non-necrotizing granulomatous inflammation with Langhans giant cells

The subretinal biopsy showed non-necrotizing granulomatous inflammation, consisting of tight aggregates of epithelioid histocytes and Langhans giant cells (Fig. 3). The lymphocytic infiltrate associated with the granulomas consisted primarily of CD3 +/CD4 + T helper cells. There was no evidence of lymphoma on light microscopy nor immunohistochemistry. Cytology on the vitreous washings showed granulomatous vitritis, with no evidence of lymphoma by flow cytometry. Microbiology did not demonstrate any growth of bacteria, mycobacteria or fungi. In the context of ophthalmic and systemic findings, the subretinal biopsy confirmed the diagnosis of ocular sarcoidosis.

**Fig. 4:**
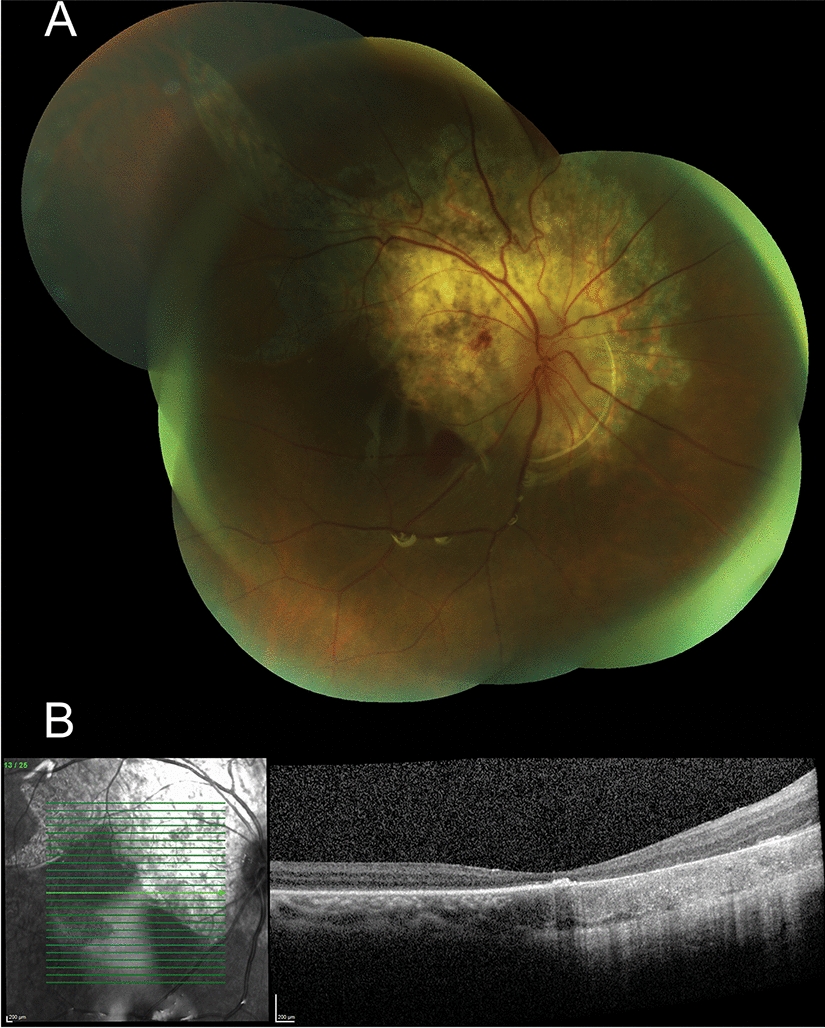
**A** Color fundus photograph of the right eye reveals peripapillary chorio-retinal atrophy in the area of the subretinal biopsy with a retinal pigment epithelial rip extending superotemporally and a small blot hemorrhage in the papillomacular bundle. **B** OCT scan shows atrophy of the outer retina at the right macula

The patient was started on oral prednisone initially at 50 mg then tapered down by 10 mg per week until cessation. He responded well to the corticosteroid regimen with resolution of macular edema. Six months following therapy, the BCVA in the right eye remained poor at 20/200. Fundus examination and OCT scans of the right eye showed a peripapillary chorioretinal atrophy and macular atrophy (Fig. 4A, B). Co-management of the systemic sarcoidosis was continued by cardiology, respiratory medicine, and immunology, with commencement of methotrexate at 10 mg weekly, given the severity of his ocular involvement.

## Discussions and conclusions

Sarcoidosis is a chronic idiopathic granulomatous inflammatory disease, that has ocular involvement in 10–80% of patients [3]. It is one of the great masquerades, along with syphilis, tuberculosis, and lymphoma. The most common ocular manifestation is granulomatous anterior uveitis. Posterior segment disease without anterior segment involvement is rare and presents a unique diagnostic challenge. Common posterior segment signs of sarcoidosis are vitritis, periphlebitis and choroidal granulomas. Peripapillary granulomas occur in 1–5% of patients with sarcoidosis and was first reported in 1964 [4, 5]. ICG imaging of sarcoid choroidal granulomas typically show hypocyanescent spots in the early and intermediate phase, which become isocyanescent or hypercyanescent in the late phase [6, 7]. Our case was particularly challenging because demonstration of granulomatous inflammation, primarily within the subretinal space has not been previously described and intraocular biopsy is uncommon in sarcoidosis. It also did not exhibit features that are typically associated with ocular sarcoidosis including bilaterality, anterior uveitis or other posterior segment signs [8].

Currently, ocular sarcoidosis is diagnosed based on a combination of intraocular signs, laboratory results and imaging findings, with or without tissue biopsies. This was formalized by the International Workshop of Ocular Sarcoidosis (IWOS), who set out objective criteria for the diagnosis of ocular sarcoidosis [8]. In the revised IWOS criteria, there are three categories, “definite ocular sarcoidosis”, where the diagnosis is supported by biopsy with compatible uveitis; “presumed ocular sarcoidosis”, no biopsy but the presence of bilateral hilar lymphadenopathy on chest x-ray or chest CT with two intraocular signs and “probable ocular sarcoidosis”, no biopsy, no hilar lymphadenopathy but three intraocular signs and two supportive systemic investigations. Prior to subretinal biopsy, our case failed to meet the requirements even for “probable ocular sarcoidosis”. There were only two possible intraocular signs consistent with the diagnostic criteria: optic disc granuloma and multiple peripheral chorioretinal lesions, but even these were only visible on ICG angiography. The only supportive systemic investigation was the slightly elevated ACE level, and this was only detected on repeat testing. The hilar lymphadenopathy was found on the PET scan but not on the chest X-ray or chest CT scan. Given the diagnostic uncertainty and worsening vision, subretinal biopsy was warranted. Recently, another international collaboration, the Standardization of Uveitis Nomenclature (SUN) Working group developed a machine learning based classification criteria for sarcoidosis-associated uveitis [9]. The SUN criteria are similar but more stringent because they only include the definite and presumed ocular sarcoidosis categories of the IWOS criteria. They found that only approximately 62% of those with “probable ocular sarcoidosis” will have sarcoidosis when a biopsy is performed and thus have omitted this category [9]. They do not include elevated serum ACE or serum lysozyme levels in their criteria due to low sensitivity levels. Despite these differences, our case has also satisfied the SUN classification criteria for sarcoidosis-associated uveitis.

In general, the most easily accessible lesion is the most preferred site for biopsy, and this commonly includes, the skin, conjunctival nodules, enlarged lacrimal glands and enlarged superficial lymph nodes. If these lesions are not present or biopsy findings are inconclusive, as in our case with the inguinal lymph node, then intrathoracic lymph nodes and/or lung parenchyma are often the next preferred options for biopsy, because they are involved in over 95% of patients and the lesions are usually accessible by flexible bronchoscopy, with minimal risk of complications [10]. In our case, hilar lymphadenopathy was not visible on the original CT chest, only identified later by the PET scan. This highlights the sensitivity of the PET scan and its usefulness in providing a range of alternative biopsy sites. Ultimately, the choice of biopsy is influenced by the presenting clinical symptoms of the organ involved and the ease and safety of the biopsy procedure. Small case series have demonstrated vitrectomy-assisted choroidal or subretinal biopsy to be safe and useful for the diagnosis of indeterminate lesions of the posterior segment [11, 12]. One such study, using 27-gauge vitrectomy to biopsy subretinal and choroidal lesions, established a diagnosis in 88.9% of eyes in lesions of 0.8 mm or larger [11]. However, with vitrectomy alone, the diagnostic yield is substantially lower, as demonstrated by Scott et al. [13] who surveyed the results of 150 patients with posterior uveitis and established a diagnosis by positive vitreous fluid analysis in only 42% of eyes. Eight of these vitreous specimens (5.3%) revealed cytopathological findings consistent with ocular sarcoidosis [13].

Determining the primary anatomical location of inflammation in peripapillary granulomas, whether it be choroidal, subretinal or the optic nerve head itself, can be difficult. A 2016 literature review conducted by Hickman et al. [14], reported 34 cases of optic nerve head granulomas, with only two having a histological diagnosis confirmed by enucleation. Interestingly, they found that the majority of cases (90.2%) had additional clinical signs of ocular sarcoidosis in the affected eyes such as keratitic precipitates, conjunctival nodules and vitritis with snowballs [14]. In 2020, a case report by Krishna et al. [15] described optic nerve head sarcoidosis mimicking an intraocular tumour. The diagnosis was only established after enucleation, with histopathological analysis revealing an extensive non-necrotizing granulomatous reaction with Langhans giant cells limited to the optic nerve itself [15]. Including their own, the authors determined that there have only been 6 previous reported cases of optic nerve head granuloma with histopathology: three enucleations, one post-mortem and two optic nerve biopsies. As far as we are aware, our case is the first with histopathology demonstrating sarcoidosis in the subretinal space.

Differential diagnoses considered for this lesion included peripapillary CNV, an unusual primary intraocular lymphoma (PIOL) and paraneoplastic cloudy vitelliform submaculopathy. Peripapillary CNV was unlikely, given the lack of leakage on the fluorescein or ICG angiogram, unconvincing response to intravitreal bevacizumab and subretinal biopsy findings. PIOL was investigated as a differential but the absence of choroidal involvement, the lack of ocular inflammation and negative haematological workup, ruled this out. This diagnostic challenge has been similarly described in a case series of nine patients who were referred with a diagnosis of PIOL but were ultimately diagnosed with ocular sarcoidosis [16]. They found that because of the high mortality with PIOL and the common assumption that most inflammation in an older patient represented a masquerade syndrome, clinicians often discounted the possibility of a primary inflammatory process. The majority of these cases eventually underwent a tissue biopsy that enabled a definitive diagnosis of sarcoidosis to be made, reinforcing the need for biopsy to exclude malignant pathology. Paraneoplastic cloudy vitelliform submaculopathy was first reported in 2014 as a subtype of ocular lymphoma [17]. These lesions are indistinct yellow subretinal deposits that are hyper-reflective and subretinal on OCT scans and appear very similar to our case. However, they are sub macular rather than centred on the optic nerve, resolve spontaneously after 3 months, and precede primary vitreoretinal lymphoma and/or primary central nervous system lymphoma within 6 months. In our case, the lesion persisted after 3 months, was not associated with the development of lymphoma elsewhere and lymphoma was excluded by cytology and flow cytometry.

In summary, ocular sarcoidosis can present as an isolated subretinal peripapillary lesion, even in the absence of more typical signs such as uveitis, vitritis or retinal vasculitis. Subretinal biopsy for histopathological confirmation can be useful in clinically equivocal cases.

## Data Availability

Not applicable.

## Abbreviations

CNV: Choroidal neovascular membrane
BCVA: Best-corrected visual acuity
OCT: Optical coherence tomography
ICG: Indocyanine green
ACE: Angiotensin-converting enzyme
MRI: Magnetic resonance imaging
CT: Computed tomography
PET: Positron emission tomography
IWOS: International Workshop of Ocular Sarcoidosis
SUN: The Standardization of Uveitis Nomenclature Working Group
PIOL: Primary intraocular lymphoma
